# Prevalence of hepatitis B, C, and D virus infection in Haiti: A systematic review and meta-analysis

**DOI:** 10.3389/fpubh.2022.1099571

**Published:** 2023-01-11

**Authors:** Jeanne Perpétue Vincent, Carolyn Nyamasege, Su Wang, Yoann Madec, Yusuke Shimakawa

**Affiliations:** ^1^Unité d'Épidémiologie des Maladies Émergentes, Institut Pasteur, Paris, France; ^2^Department of Health and Human Services, Institute for Health Policy and Practice, University of New Hampshire, Concord, NH, United States; ^3^Viral Hepatitis Program, Cooperman Barnabas Medical Center, Livingston, NJ, United States

**Keywords:** Haiti, hepatitis B, hepatitis C, hepatitis D, prevalence, epidemiology, elimination

## Abstract

**Background:**

Viral hepatitis causes an important global health burden. In 2016, the World Health Assembly adopted an objective to globally eliminate this as a public health threat by 2030. However, significant gaps exist between countries in their progress. Haiti is the last country that has introduced infant hepatitis B vaccines into the routine immunization program in the Region of the Americas, and its schedule still does not incorporate birth dose vaccines. As the first step to raise awareness of viral hepatitis in this country, we conducted a systematic review and meta-analysis to estimate the prevalence of hepatitis B (HBV), C (HCV), and D (HDV) viruses in Haiti.

**Methods:**

We searched PubMed, EMBASE, Web of Science and Scopus for studies reporting the prevalence of HBV, HCV and HDV among Haitian, with no language restriction, published until November 30th, 2021. Prevalence was pooled *via* a random-effects meta-analysis using a generalized linear mixed model with the logit link.

**Results:**

Of 453 articles retrieved, 25 studies were included: 16 reported the prevalence of hepatitis B surface antigen (HBsAg), three for anti-HCV antibody, and six for both HBsAg and anti-HCV. No study was found for HDV prevalence. The pooled prevalence of HBsAg was 0.7% [95% confidence interval (CI): 0.3–1.4, *I*^2^ = 77.7%] among children, 3.5% (95% CI: 2.8–4.4, *I*^2^ = 93.2%) in the general adult population and 7.4% (95% CI: 4.0–13.3, *I*^2^ = 83.9%) in high-risk adult population. The pooled prevalence of anti-HCV antibody was 0.9% (95% CI: 0.6–1.4, *I*^2^ = 93.5%) among the general population and 1.4% (95% CI: 0.4–4.2, *I*^2^ = 0.0%) in high-risk adult population. No study reported the prevalence of anti-HCV antibody exclusively in children.

**Interpretation:**

The prevalence of blood-borne hepatitis, particularly that of HBV, is substantial in Haiti. The introduction of birth dose hepatitis B vaccines and improving access to testing and treatment services should be urgently considered to meet the elimination goal.

**Systematic review registration:**

https://www.crd.york.ac.uk/prospero/display_record.php?ID=CRD42022298081, identifier: PROSPERO (CRD42022298081).

## Background

An estimated 354 million people live with hepatitis B (HBV) or hepatitis C virus (HCV) worldwide, and among them more than one million die yearly from complications such as hepatocellular carcinoma or cirrhosis (1). The majority of this burden is borne by people in Africa and Asia. In the Americas, the number of people chronically infected with HBV and HCV were estimated at 14 million (2). The number of deaths related to HBV and HCV in 2019 for the region were estimated at 15,000 and 31,000, respectively (1).

As these chronic viral infections progress asymptomatically over the years until serious liver damage is established, most people chronically infected with HBV or HCV remain undiagnosed unless there is a screening program for these infections targeting asymptomatic people. In fact, The World Health Organization (WHO) estimates that only 10% of people living with HBV and 21% of those living with HCV have been diagnosed (1). The WHO presently aims to achieve the worldwide elimination of hepatitis B and C as public health threat by 2030 (3).

Haiti is a small country of 11 million people located in the Caribbean, where little attention has been paid to viral hepatitis. The country was the last in the Americas to introduce hepatitis B vaccines into the routine infant immunization program in 2012 using pentavalent vaccines scheduled at 6, 10, and 14 weeks of life (4). In 2020, 8 years after the introduction of the hepatitis B vaccines, coverage of three-dose pentavalent vaccines still remained low at 51% among 1-year-old children according to the WHO/UNICEF data (5). Moreover, the birth dose vaccination is not yet introduced into the national immunization program and pregnant women are not systematically screened for HBV to prevent mother-to-child transmission (MTCT) despite the WHO's recommendations (6). There is no national program targeting chronic viral hepatitis in Haiti.

To date, there is no systematic review specific to Haiti to estimate the prevalence of chronic viral hepatitis in this country. Regarding the prevalence of HBsAg, studies on a global or regional scale have provided country-specific estimates including that of Haiti, but with marked variations between studies. In a systematic review and meta-analysis by Schweitzer et al. (7) published in 2015, an estimated 13.6% (95% CI: 9.0–19.9) of Haitians were positive for HBsAg. Kowdley et al. also conducted a systematic review and meta-analysis, and reported a pooled prevalence of 4.8% (95% CI: 3.9–5.7) in their first publication in 2011 and 4.6% (95% CI: 3.2–6.0) in their updated publication in 2021 (8, 9). On the basis of a combination of literature review and expert opinions, the Polaris Observatory Collaborators estimated that the prevalence of HBsAg in Haiti in 2016 was 2.9% (95% CI: 2.7–4.1) (10). Most recently, the Global Burden of Disease (GBD) study reported a decrease in prevalence of HBV from 1.9% (95% CI: 1.5–2.2) in 1990 to 1.6% (95% CI: 1.3–1.9) in 2019, using a combination of systematic review and mathematical modeling (11). Interpretation of these heterogeneous estimates is complicated. Moreover, besides the articles by Kowdley et al., these estimations were based on a small number of studies (two for Schweitzer et al., four for Polaris, and one for GBD). Regarding HCV and hepatitis D virus (HDV), none of the previous systematic reviews estimating the global prevalence of these infections included a study from Haiti (12–15).

We, therefore, conducted a systematic review and meta-analysis specific to Haiti for the prevalence of HBV, HCV, and HDV among three groups: (i) children, (ii) the general adult population, and (iii) the high-risk adult population. The data generated by this work can serve as baseline data before any public health interventions targeting these infections are implemented in this country.

## Methods

### Search strategy and eligibility criteria

This systematic review was conducted following a protocol registered in PROSPERO (CRD42022298081) and reported according to the PRISMA guidelines (16). Four databases (PubMed, EMBASE, Web of Science and Scopus) were searched from their inception until November 30, 2021, without language restriction. Following search terms and their respective variations were used: “Haiti” AND “(“HBV” OR “HCV” OR “HDV”)” (detailed search strategy in Supplementary material). References of included studies were also manually searched. Original articles and conference abstracts of any study design were eligible if they reported the numerator (number with a positive result) and the denominator (number who had a test) enabling us to calculate the prevalence of viral hepatitis markers in individuals of any age living in Haiti or immigrants of Haitian origin (i.e., those born in Haiti and residing in other countries). Gray literature, including government reports or WHO reports, was also eligible if it provided these data. We primarily considered the following markers: HBsAg, anti-HCV antibody, and anti-HDV antibody. In addition, as secondary outcomes we considered the following: HBV DNA and hepatitis B e antigen (HBeAg) in people positive for HBsAg, HCV RNA in people positive for anti-HCV, and HDV RNA in people positive for anti-HDV antibody. Clinical definition of chronic HBV infection is the persistence of HBsAg for at least 6 months (17). In contrast, for an epidemiological study in a country with high HBV endemicity, positive HBsAg at a single time point can be reasonably considered as a chronic HBV infection (18).

### Data selection

Titles and abstracts of all the articles identified through the search strategy were independently screened for relevance by two reviewers (JPV and CN). Following the selection of potentially eligible articles through the screening, a full-text reading was performed to review and extract the data. Any discrepancies between two reviewers were resolved through discussion within the team. A standardized form was edited to extract, by the same two reviewers, the following data from each study: first author's name, year of publication, journal name, language used to report the study, study setting, type of study population, period of data collection, participants' baseline characteristics, inclusion and exclusion criteria, type of assay used for viral hepatitis markers, total number of people tested for each of viral hepatitis markers (i.e., denominators), and total number of people tested positive for each of viral hepatitis markers (i.e., numerators).

The study population was classified into three groups: children, the general adult population and the high-risk adult population. We defined adults as those aged ≥15 years. The high-risk adult population includes people living with other comorbidities (e.g., HIV, tuberculosis) as well as refugees, and those with symptoms related to liver disease (e.g., ascites). The general adult population was defined as those not presenting the characteristics defined above as high risk. Whenever possible, population-specific estimates were obtained in a study that recruited different groups of people.

Whenever information was missing in the full-text paper that limits our ability to make a final decision on whether to include the study, corresponding authors were systematically contacted by e-mail. The risk of bias was evaluated using a framework introduced by Hoy et al. (19).

### Data analysis

The outcomes of interest were as below: the prevalence of HBsAg, the prevalence of HBeAg or positive HBV DNA in HBsAg-positive individuals, the prevalence of anti-HCV antibody or HCV RNA, the prevalence of anti-HDV antibody or HDV RNA in people chronically infected with HBV. The prevalence of each marker was computed by dividing the number of people tested positive by the total number of people tested. Meta-analysis was carried using the “metaprop” command with R 4.2.2 (R Foundation for Statistical Computing, Vienna, Austria) and RStudio Desktop 2022.07.2 (Rstudio, PBC, Boston, MA, USA) (20). Proportions were pooled *via* a random-effects meta-analysis using a generalized linear mixed model (GLMM) with a logit link approach. The percentage of total variation between studies due to heterogeneity was evaluated using the *I*^2^ statistic. Subgroup analyses were conducted by place of residence at the time of testing (Haiti or outside), and by recruitment period (before or after the year 2000) among the general adult population. We also analyzed the subgroups of pregnant women and people living with HIV (PLHIV).

## Results

**Figure 1:**
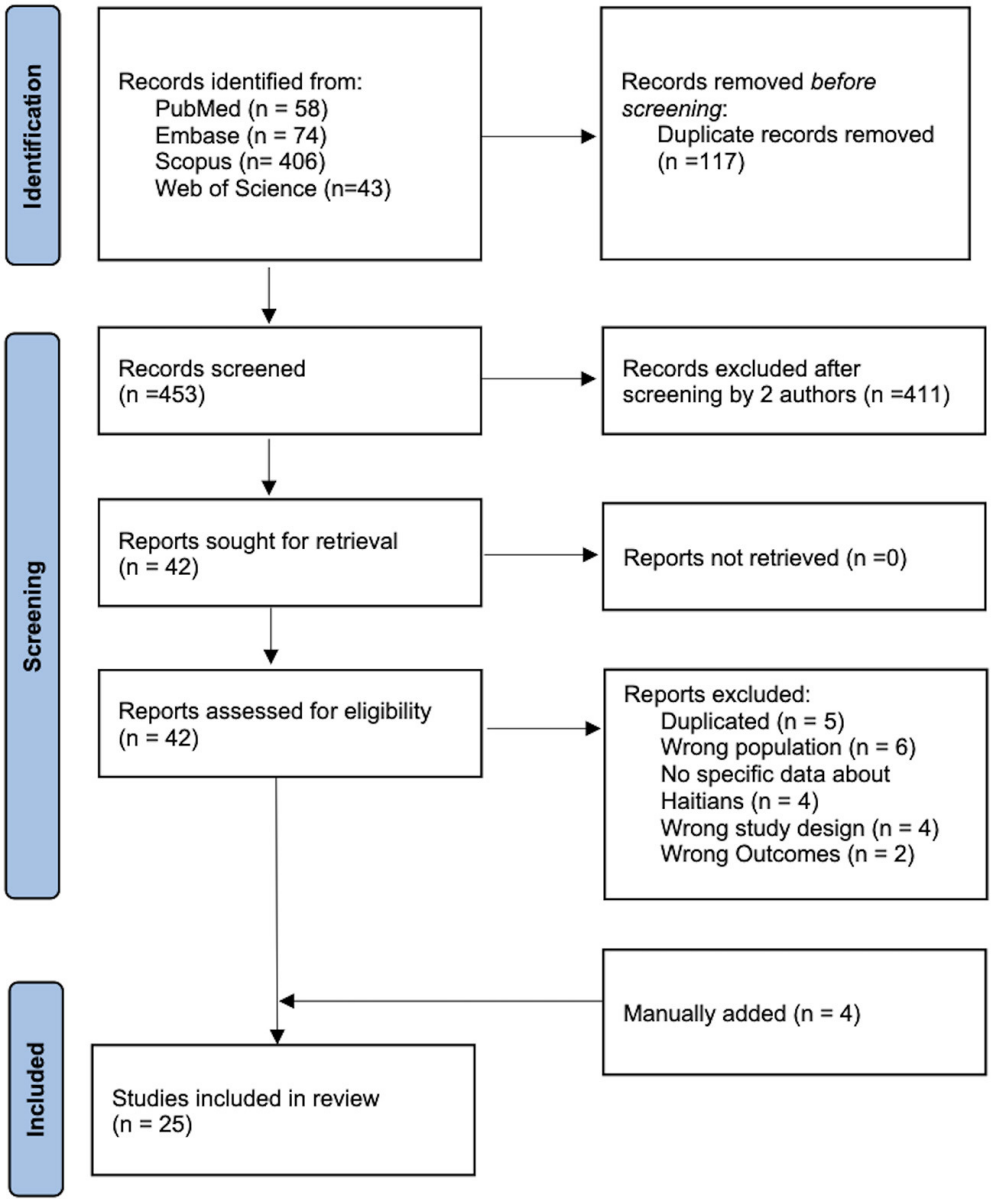
PRISMA flow diagram showing the study selection process.

A total of 453 articles were identified after the removal of duplicates. Following the independent screening by two reviewers, 42 full-text articles were assessed for eligibility, and 21 met the eligibility criteria. In addition, four more eligible articles were manually identified from the references of the included studies and other review articles. Finally, 25 studies were included in the meta-analysis for the following hepatitis markers: HBsAg only (16 studies), anti-HCV antibody only (three studies), and both HBsAg and anti-HCV antibody (6 studies). There was no study assessing HDV prevalence among Haitian (Figure 1).

Of the 22 studies reporting the prevalence of HBsAg, six were classified as having a low risk of bias, seven as a moderate, and nine as a high risk. Of the nine studies reporting the prevalence of anti-HCV antibody, two were classified as a low risk of bias, six as moderate, and one as high. There was a wide variation in the recruitment sites. For HBsAg, seven studies recruited participants in hospitals, seven during antenatal or perinatal care, two from blood bank, and six in community survey. For anti-HCV antibody, four studies recruited in hospital, two during antenatal care, two from blood bank, and three in community survey.

### HBV prevalence

Twenty studies evaluated adult population, including two with a mixed population of adults and children, and two studies exclusively evaluated children (Table 1). Eleven studies were conducted on people residing in Haiti, nine on immigrants and two on refugees. Of 20 adult studies, 15 reported prevalence in a single population and five reported prevalence in separate groups of people. Consequently, a total of 27 estimates were derived: 19 for the general adult population (including four for blood donors and seven for women during antenatal or perinatal care visit), and eight for the high-risk adult population (including four estimates for PLHIV, one for patients with tuberculosis, two for refugees, and one for patients with ascites). The characteristics of the studies reporting HBsAg prevalence were summarized in Table 1.

**Table 1:** Studies reporting the prevalence of HBsAg among Haitians.

**Authors, reference**	**Country**	**Study design**	**Study setting**	**Population**	**Adults/ children**	**Mean age ±SD (years)**	**Type of HBsAg assay**	**Male sex (%)**	**HIV rate (%)**	**No. tested**	**No. positive**	**Prevalence (%)**
Ollé-Goig (21)	Haiti	Case-control	Hospital	Patients with ascites	Adults	37.0	RIA	31.6	N/R	19	6	31.6
				Control patients (no ascites)	Adults	36.9	RIA	30.8		39	2	5.1
Malison et al. (22)	USA	Cross-sectional	Community	Immigrants (mothers of children up to 10 years old)	Adults	N/R	RIA	0.0	N/R	51	2	3.9
Delage et al. (23)	Canada	Cross-sectional	Maternity ward	Immigrants (pregnant women)	Adults	N/R	RIA or EIA	0.0	N/R	543	18	3.3
Gordon et al. (24)	USA	Cross-sectional	Hospital	Immigrants (HIV positive)	Adults	N/R	RIA	N/R	100	15	2	13.3
Jonas et al. (25)	USA	Cross-sectional	Maternity ward	Immigrants (pregnant women)	Adults	N/R	RIA	0.0	N/R	606	23	3.8
Lange et al. (26)	USA	Cross-sectional	Immigration center	Refugee (boat people)	Adults	27.7	RIA	100	7.0	171	21	12.3
Schill et al. (27)	Haiti	Cross-sectional	ANC and hospital	Pregnant women and outpatients	Adults and children	31.3	EIA	25.9	5.2	115	15	13.0
Rosenblum et al. (28)	USA	Cross-sectional	Community	Immigrants	Adults and children	36.0	RIA	50.0	12.0	117	5	4.3
Boulos et al. (29)	Haiti	Case-control	ANC	Pregnant women (HTLV positive)	Adults	28.0 ± 5.4	RIA	0.0	0.0	45	1	2.2
				Pregnant women (HIV positive)	Adults	25.6 ± 5.4	RIA	0.0	100	95	7	7.4
				Pregnant women	Adults	25.4 ± 5.4	RIA	0.0	0.0	89	6	6.7
Andernach et al. (30)	Haiti	Cross-sectional	ANC	Pregnant women	Adults	N/R	EIA	0.0	N/R	7,147	357	5.0
Exantus and Dall'Amico (31)	Haiti	Cross-sectional	Hospital	Patients with nephrotic syndrome	Children	6.4	N/R	55.4	5.3	17	1	5.9
Rein et al. (32)	USA	Cross-sectional	Immigration center^§^	Refugee	N/R	N/R	N/R	N/R	N/R	659	17	2.6
Tohme et al. (4)	Haiti	Cross-sectional	ANC	Pregnant women (HIV positive)	Adults	N/R	CLIA	0.0	100	123	3	2.4
				Pregnant women					0.0	1,184	30	2.5
Ngongondo et al. (33)	Haiti	RCT	Hospital	Patients with HIV	Adults	36.9 ± 8.9	N/R	48.6	100	102	9	8.8
Jean-Baptiste et al. (34)	Haiti	Cross-sectional	Blood bank	Blood donors	Adults	N/R	EIA	N/R	N/R	198,758	7,553	3.8
Childs et al. (35)	Haiti	Cross-sectional	Community	General population	Children	6.0	RDT	49.8	N/R	1,152	7	0.6
Izquierdo et al. (36)	Chile	Cohort	ANC	Immigrants (pregnant women)	Adults	N/R	CLIA	0.0	N/R	829	31	3.7
Fuster et al. (37)	Chile	Cross-sectional	Community	Immigrants	Adults	31.4 ± 7.6	CMIA	39.6	2.4	468	16	3.4
PAHO (38)	Haiti	Cross-sectional	Blood bank	Blood donors (2015)	Adults	N/R	N/R^*^	N/R	0.8	27,752	1,022	3.7
				Blood donors (2016)					0.6	25,699	838	3.3
				Blood donors (2017)					0.5	28,018	657	2.3
Brogden et al. (39–41)	USA	Cross-sectional	Community	Immigrants	Adults	49.0	EIA	38.0	N/R	21	1	4.8
			Hospital	Immigrants	Adults	53.6 ± 18.2	EIA	37.2		3,275	50	1.5
Franke et al. (42)	Haiti	Cohort	Hospital	Patients with tuberculosis	Adults	33 (median) (IQR: 28–39)	RDT	50.0	37.5	16	1	6.3
Jones et al. (43)	USA	RCT	Community	Immigrants	Adults	58.5 ± 4.1	EIA	25.0	N/R	53	0	0.0

^*^EIA confirmed by CMIA if the type of assay is unchanged from the report by Jean-Baptiste et al. (34).

^§^Data based on report from state refugee health coordinators.

ANC, antenatal clinic; CLIA, chemiluminescence immunoassay; CMIA, chemiluminescence microparticle immunoassay; EIA, enzyme immunoassay; HBsAg, hepatitis B surface antigen; HIV, human immunodeficiency virus; HTLV, human T-cell lymphotropic virus; N/R, not reported; PAHO, Pan American Health Organization; RCT, randomized controlled trial; RDT, rapid diagnostic test; SD, standard deviation.

A total of 297,178 individuals were assessed for HBsAg. In children, the pooled prevalence was 0.7% (95% CI: 0.3–1.4, *I*^2^ = 77.7%). Exantus et al. (31) studied a group of children with nephrotic syndrome evaluated from 1990 to 2008, before the introduction of HBV vaccines in the national program, while Childs et al. (35) conducted a nationally representative community-based survey in 2017 among 5–7 year-olds, including both children born before and after the hepatitis B immunization program.

**Figure 2:**
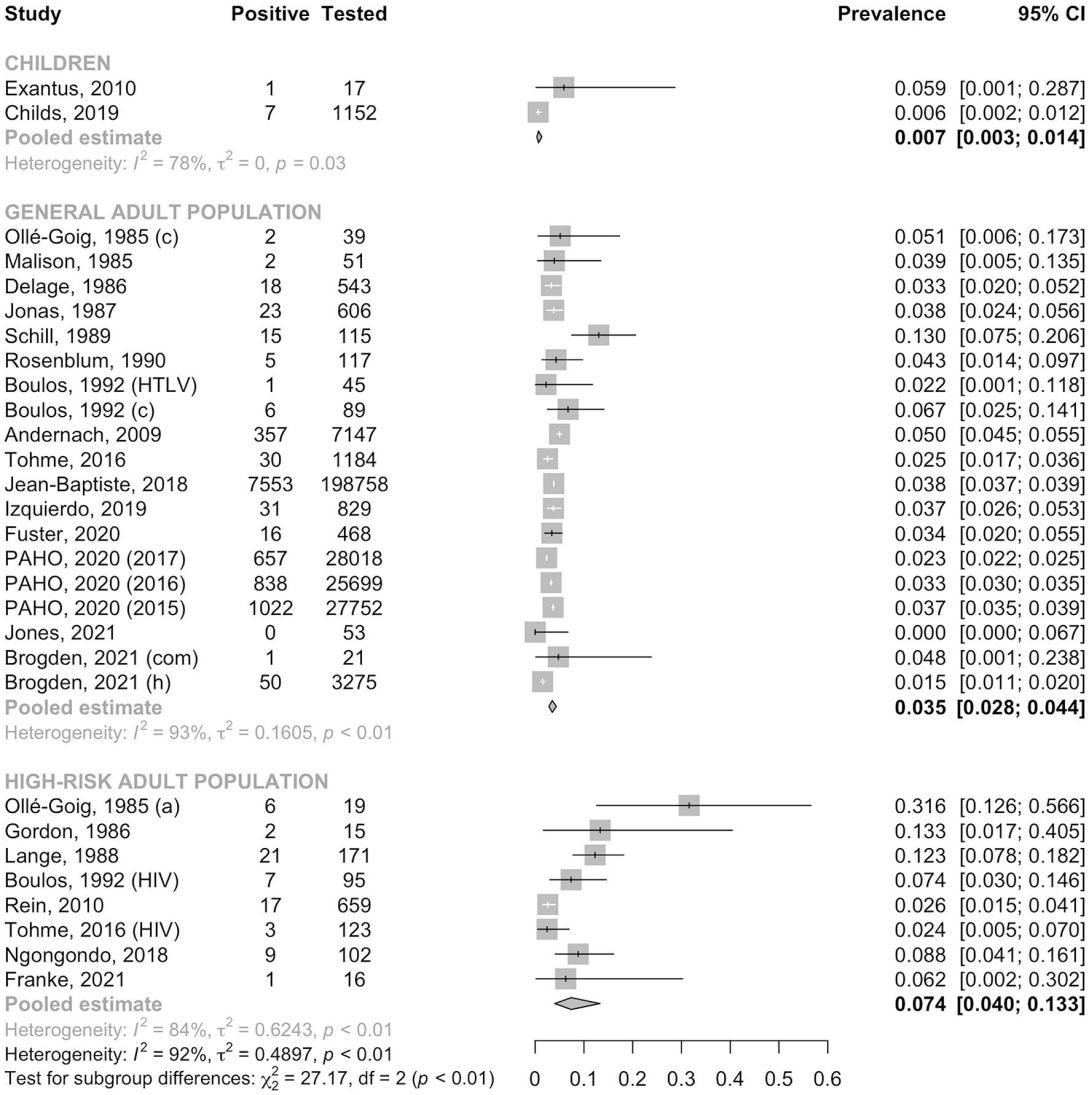
Prevalence of HBsAg in children, the general adult population and the high-risk adult population. a, ascites group; c, control group; com, community recruitment; h, hospital recruitment; HIV, human immunodeficiency virus positive group; HTLV, human T-cell lymphotropic virus positive group; PAHO, Pan American Health Organization.

**Figure 3:**
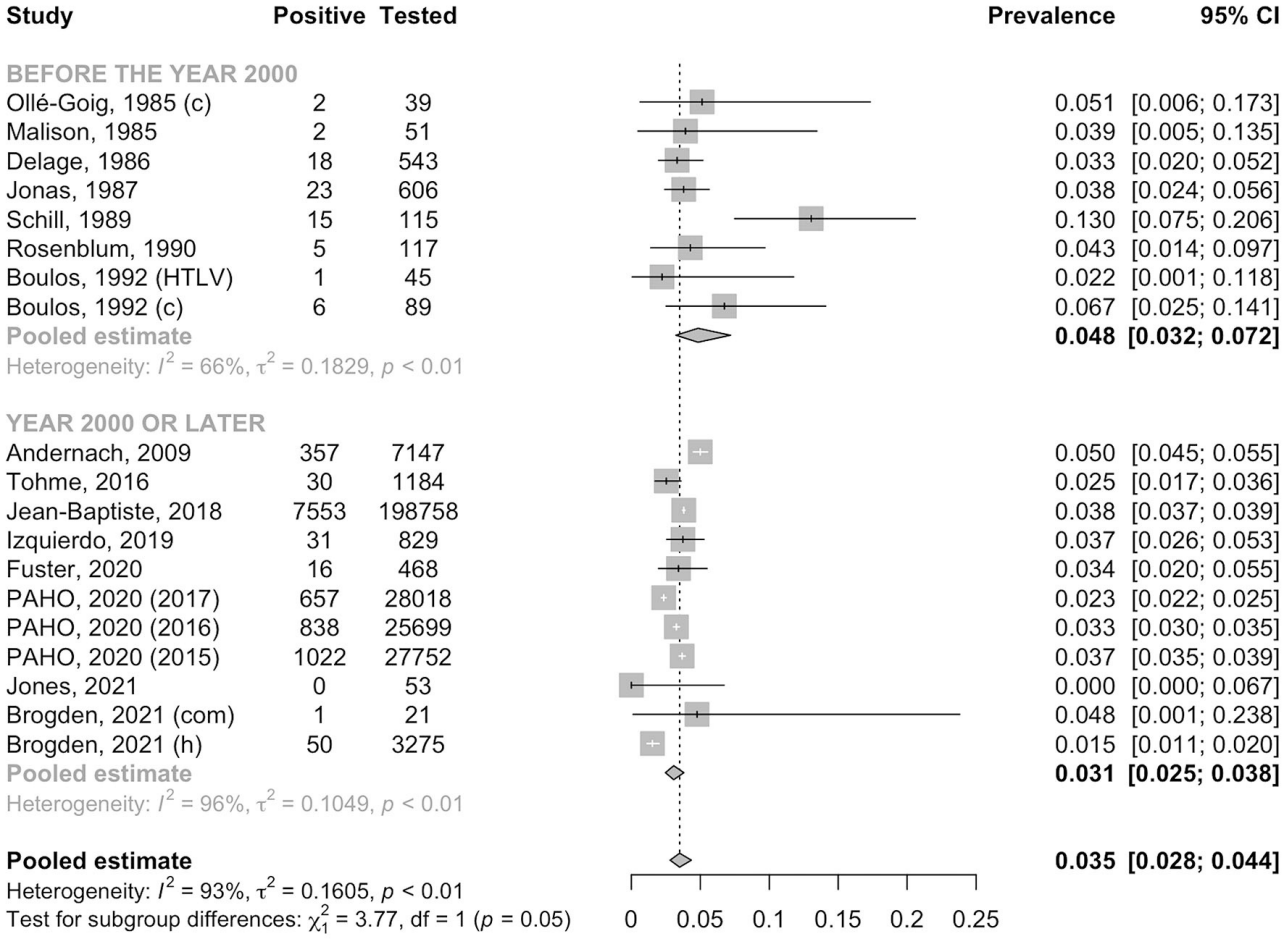
Prevalence of HBsAg in the general adult population by year. c, control group; com, community recruitment; h, hospital recruitment; HTLV, human T-cell lymphotropic virus positive group; PAHO, Pan American Health Organization.

**Figure 4:**
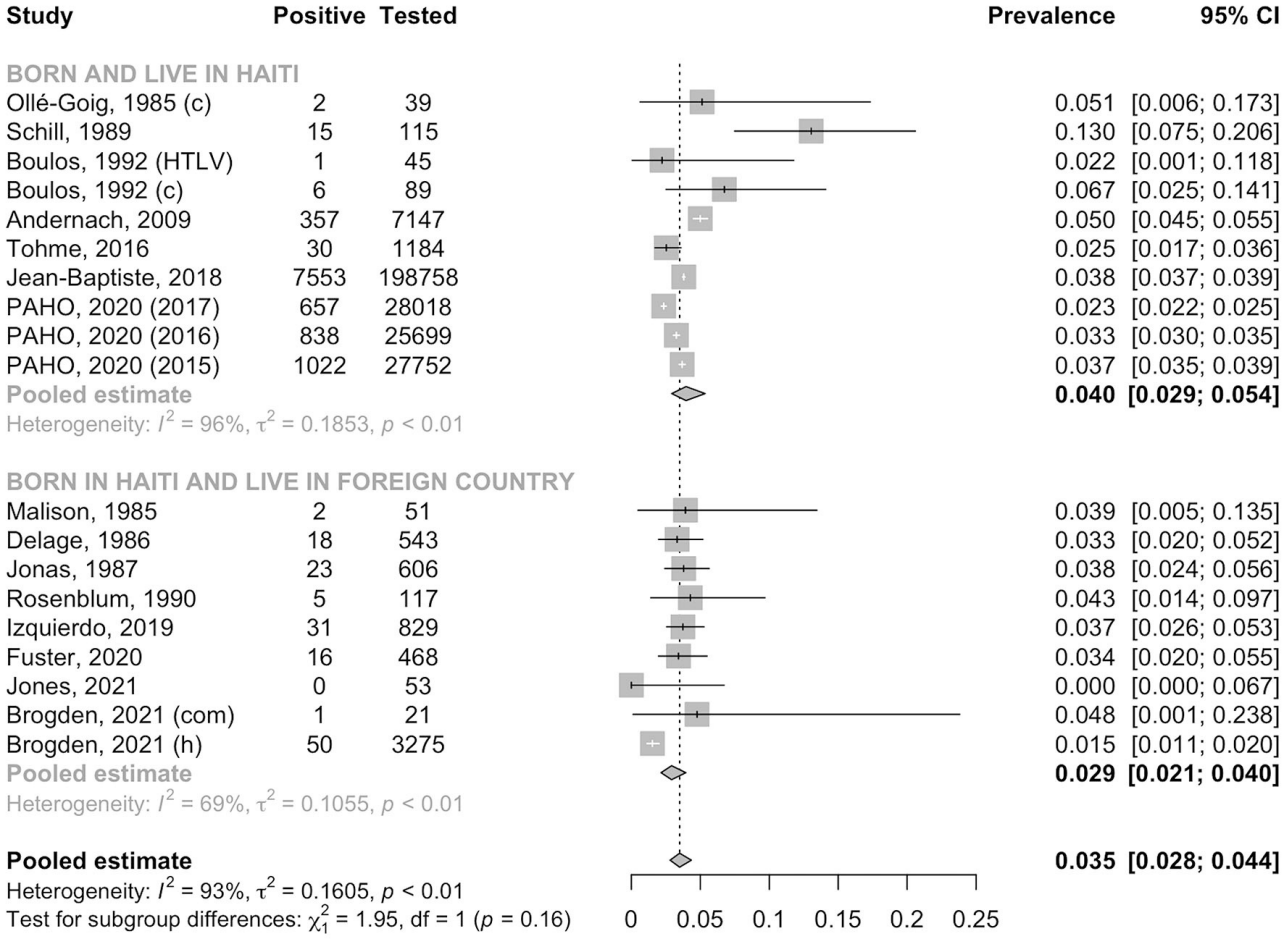
Prevalence of HBsAg in the general adult population by place of residence. a, ascites group; c, control group; com, community recruitment; h, hospital recruitment; HIV, human immunodeficiency virus positive group; HTLV, human T-cell lymphotropic virus positive group; PAHO, Pan American Health Organization.

**Figure 5:**
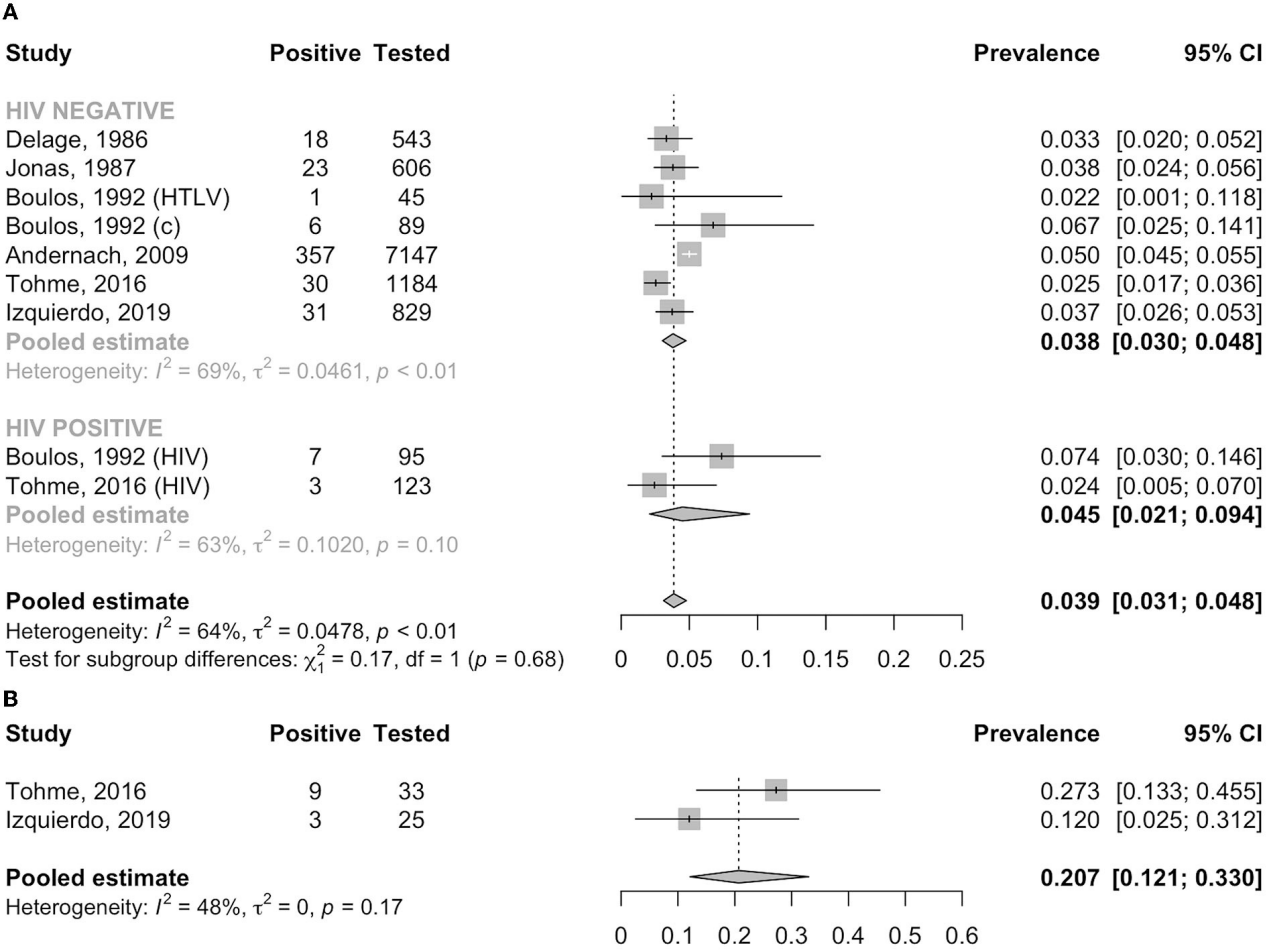
**(A)** Prevalence of HBsAg in pregnant women by HIV status. c, control group; HIV, human immunodeficiency virus positive group; HTLV, human T-cell lymphotropic virus positive group. **(B)** Prevalence of high viral load (≥200,000 IU/ml) in HBsAg-positive pregnant women.

In the general adult population, the pooled prevalence of HBsAg was 3.5% (95% CI: 2.8–4.4, *I*^2^ = 93.2%); this was significantly lower than that in high-risk populations (7.4%, 95% CI: 4.0–13.3, *I*^2^ = 83.9%, *p* for heterogeneity between groups < 0.001, Figure 2). In the general adult population, the prevalence tended to be lower in studies conducted after the year 2000 (3.1%, 95% CI: 2.5–3.8, *I*^2^ = 95.8%) than in those conducted before 2000 (4.8%, 95% CI: 3.2–7.2, *I*^2^ = 66.4%, *p* for heterogeneity between groups = 0.05, Figure 3). The prevalence in the general adult population did not significantly vary according to their place of residence at the time of testing: 4.0 % (95% CI: 2.9–5.4, *I*^2^ = 96.0%) in those recruited in Haiti and 2.9% (95% CI: 2.1–3.9, *I*^2^ = 69.5%) in immigrants of Haitian origin (*p* for heterogeneity between groups = 0.16, Figure 4). In pregnant women, no difference was observed in the prevalence between those not infected with HIV (3.8%, 95% CI: 3.0–4.8, *I*^2^ = 69.1%) and those infected with HIV (4.5%, 95% CI: 2.1–9.4, *I*^2^ = 63.0%, *p* for heterogeneity between groups = 0.68) (Figure 5A). In PLHIV the prevalence was estimated at 6.3% (95% CI: 3.7–10.5, *I*^2^ = 39.6%) (Supplementary Figure 1).

Sixteen studies only tested HBsAg as the sole marker of HBV, three studies tested HBsAg and HBeAg (22–24), two studies tested HBsAg and HBV DNA (4, 30), and one study tested both HBeAg and HBV DNA in addition to HBsAg (36). In four studies reporting HBeAg sero-status, two studied pregnant women (23, 36), one studied mothers of children up to 10 years of age (22), and one studied PLHIV (24). Delage et al. and Malison et al. found none of HBsAg-positive women were positive for HBeAg (0/32 and 0/2, respectively) (22, 23). In contrast, Izquierdo et al. found that 12.9% (4/31) of the HBsAg-positive pregnant women carried HBeAg (36). The pooled prevalence of HBeAg among HBsAg-positive mothers was 6.7% (95% CI: 1.0–34.2, *I*^2^ = 0.0%) (Supplementary Figure 2). In PLHIV, Gordon et al. identified two HBeAg-positive participants out of two HBsAg-positive (24).

Three studies reported HBV DNA PCR test results in people identified to carry HBsAg (4, 30, 36). Tohme et al. (4) detected HBV DNA among 78.8% (26/33) of HBsAg-positive pregnant women, with nine (27.3%) having viral load >200,000 IU/ml using an in-house quantitative PCR with a lower limit of detection of 50 IU/ml. Izquierdo et al. (36) detected HBV DNA in 92.0% (23/25) of HBsAg-positive pregnant women using the Abbott RealTime HBV assay with a lower limit of quantitation of 10 IU/ml; three (12.0%) women had viral load ≥200,000 IU/ml. The pooled prevalence of HBV DNA viral load ≥200,000 IU/ml among pregnant women was 20.7% (95% CI: 12.1–33.0, *I*^2^ = 48.1%) (Figure 5B).

Andernach et al. detected HBV DNA in 77.2% (247/320) of HBsAg-positive pregnant women by an in-house PCR. The later was the only study that analyzed HBV genotypes and reported that the majority (71.5%, 128/179) of the infections were caused by HBV genotype A (including A1, A2, and A5), followed by D (22.3%, 40/179; including D3 and D4) and E (6.1%, 11/179) (30).

### HCV prevalence

The characteristics of the nine studies reporting anti-HCV antibody prevalence are presented in Table 2. Seven studies exclusively recruited adults while two recruited both adults and children. Five studies were conducted in people residing in Haiti and four in immigrants. The nine adults studies provided 13 estimates: 11 for the general adult population (including four groups of blood donors, one group of pregnant women during antenatal care, three groups of adults recruited in community settings and three from hospitals among outpatients, surgery patients and those attending emergency room) and two for the high-risk adult population (including one with outpatients presenting AIDS suggestive symptoms and another comprising patients with tuberculosis).

**Table 2:** Studies reporting the prevalence of anti-HCV antibody among Haitians.

**Authors, reference**	**Country**	**Study design**	**Study setting**	**Population**	**Adults/ children**	**Mean age ±SD (years)**	**Type of anti-HCV antibody assay**	**Male sex (%)**	**HIV rate (%)**	**No. tested**	**No. positive**	**Prevalence (%)**
Allain et al. (44)	Haiti	Cross-sectional	Hospital	Patients with AIDS suggestive symptoms	Adults and children	(Range: 2–72)	EIA	39.3	39.3	200	3	1.5
			ANC	Pregnant women	Adults	25.0 (median) (range: 15–49)	EIA	0.0	4.0	500	2	0.4
			Hospital	Surgery patients	Adults and children	(Range: 1–83)	EIA	46.9	6.1	228	2	0.9
Talarmin et al. (45)	French Guiana	Cross-sectional	Community and ANC	Immigrants	Adults	N/R	EIA	N/R	N/R	66	1	1.5
Hepburn et al. (46)	Haiti	Cross-sectional	Hospital	Outpatients with laboratory tests	Adults	33.7 ± 15.9	EIA	67.0	N/R	500	22	4.4
Jean-Baptiste et al. (34)	Haiti	Cross-sectional	Blood bank	Blood donors	Adults	N/R	EIA	N/R	N/R	198,758	1,113	0.6
Fuster et al. (37)	Chile	Cross-sectional	Community	Immigrants	Adults	31.4 ± 7.6	CMIA	39.6	2.4	468	1	0.2
PAHO (38)	Haiti	Cross-sectional	Blood bank	Blood donors (2015)	Adults	N/R	N/R^*^	N/R	0.8	27,752	236	0.9
				Blood donors (2016)					0.6	25,699	175	0.7
				Blood donors (2017)					0.5	28,018	239	0.6
Brogden et al. (39–41)	USA	Cross-sectional	Hospital	Immigrants	Adults	53.6 ± 18.2	EIA	37.2	N/R	2,320	25	1.1
Franke et al. (42)	Haiti	Cohort	Hospital	Patients with tuberculosis	Adults	33 (median) (IQR: 28–39)	RDT	50.0	37.5	16	0	0.0
Jones et al. (43)	USA	RCT	Community	Immigrants	Adults	58.5 ± 4.1	RDT	25.0	N/R	56	2	3.6

^*^EIA confirmed by CMIA if the type of assay is unchanged from the report by Jean-Baptiste et al. (34).

AIDS, acquired immunodeficiency syndrome; ANC, antenatal clinic; CMIA, chemiluminescence microparticle immunoassay; EIA, enzyme immunoassay; HCV, hepatitis C virus; HIV, human immunodeficiency virus; N/R, not reported; PAHO, Pan American Health Organization; RCT, randomized controlled trial; RDT, rapid diagnostic test; SD, standard deviation.

**Figure 6:**
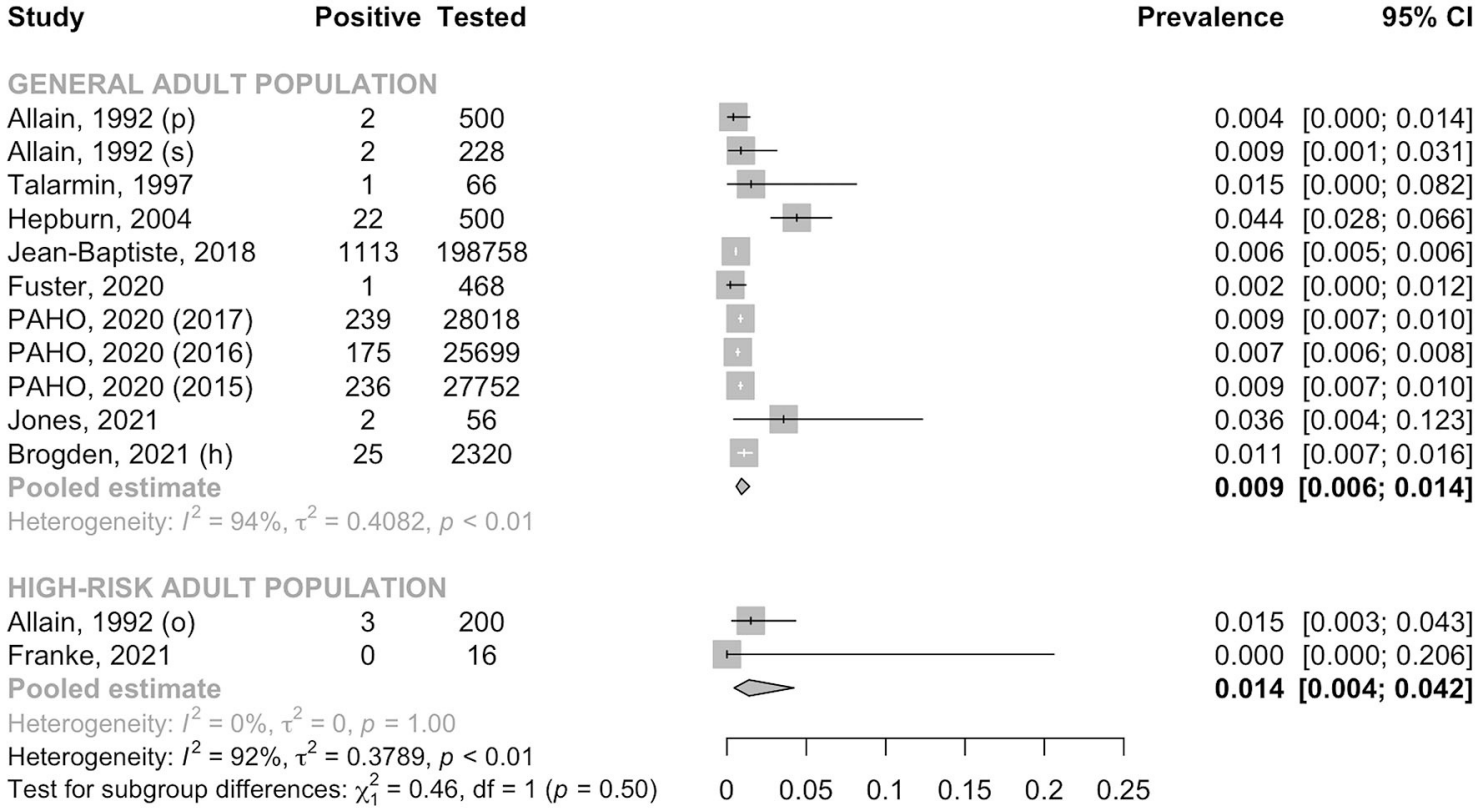
Prevalence of anti-HCV antibody in the general adult population and the high-risk adult population. h, hospital recruitment; o, outpatient; p, pregnant women; PAHO, Pan American Health Organization; s, surgery patient.

**Figure 7:**
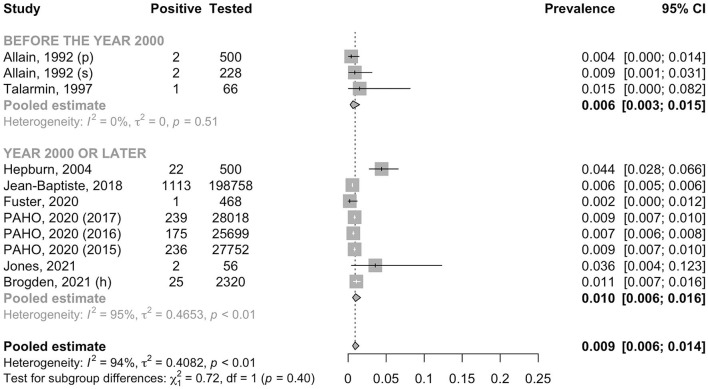
Prevalence of anti-HCV antibody in the general adult population by year. h, hospital recruitment; p, pregnant women; PAHO, Pan American Health Organization; s, surgery patient.

**Figure 8:**
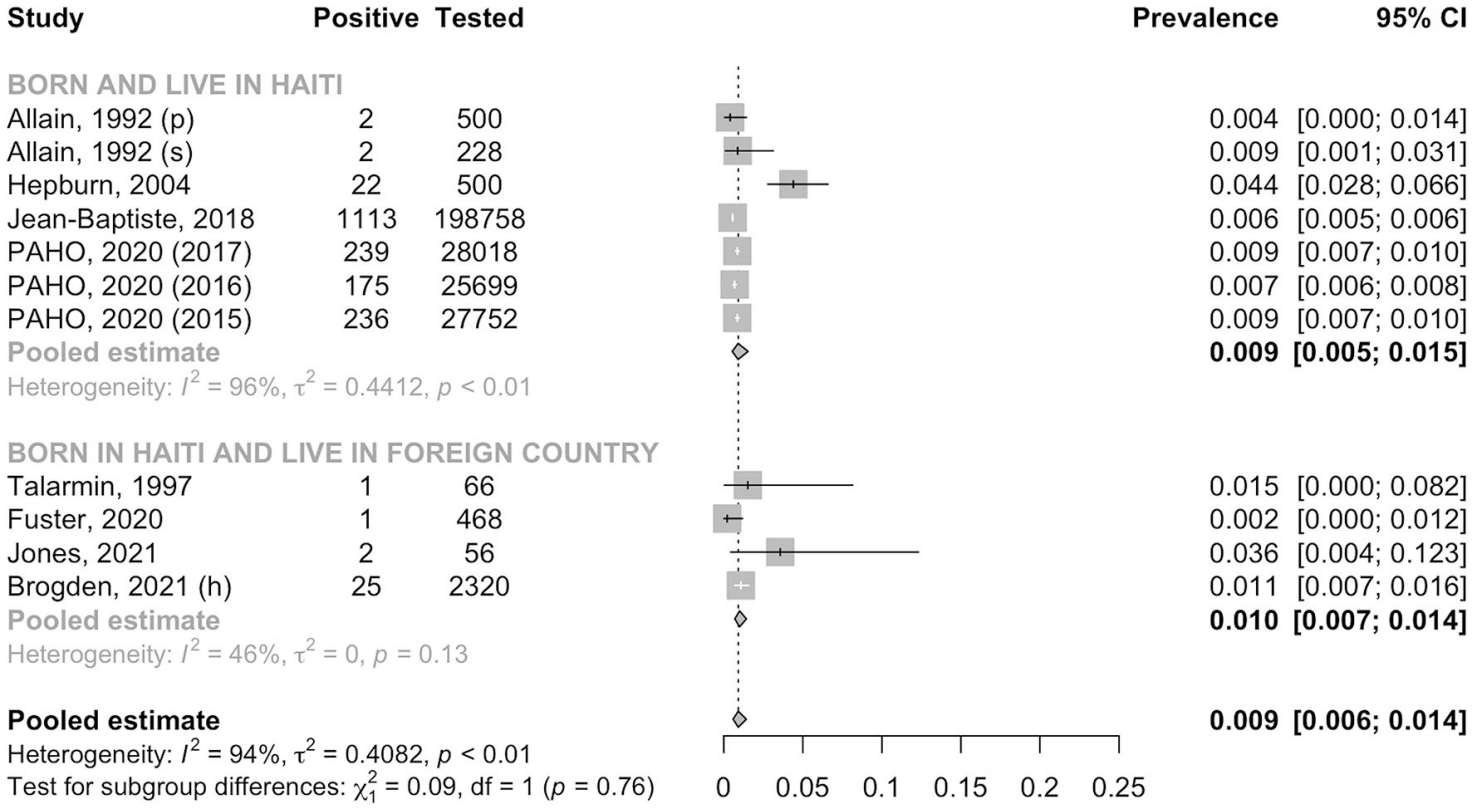
Prevalence of anti-HCV antibody in the general adult population by place of residence. h, hospital recruitment; p, pregnant women; PAHO, Pan American Health Organization; s, surgery patient.

A total of 284,581 individuals were assessed for anti-HCV antibody. The pooled prevalence was 0.9% (95% CI: 0.6–1.4, *I*^2^ = 93.5%) in the general adult population and 1.4% (95% CI: 0.4–4.2, *I*^2^ = 0.0%) in the high-risk group (*p* for heterogeneity between groups = 0.50) (Figure 6). In the general adult population, there was no evidence for the difference in the prevalence between the studies conducted before the year 2000 (0.6%, 95% CI: 0.3–1.5, *I*^2^ = 0.0%) and after 2000 (1.0%, 95% CI: 0.6–1.6, *I*^2^ = 95.4%) (*p* for heterogeneity between groups = 0.40) (Figure 7). Similarly, there was no evidence for the difference in the prevalence in the general adult population between those residing in Haiti and in foreign countries at the time of testing (0.9%, 95% CI: 0.5–1.5, *I*^2^ = 95.7% and 1.0%, 95% CI: 0.7–1.4, *I*^2^ = 46.5%, respectively) (*p* for heterogeneity between groups = 0.76) (Figure 8).

HCV RNA results were available in two studies (37, 39). Of 468 participants screened for anti-HCV, Fuster at al. (37) identified one participant positive for anti-HCV; HCV RNA was not detected in this anti-HCV positive individual using a Roche COBAS Real-Time PCR. In 25 participants positive for anti-HCV, Brogden et al. (39) reported detectable HCV RNA in ten (40.0%) using Abbott Alinity m reverse transcription PCR.

## Discussion

Through a systematic review and meta-analysis, the pooled prevalence of HBsAg was 3.5% (95% CI: 2.8–4.4) and that of anti-HCV was 0.9% (95% CI: 0.6–1.4) among the general adult population from Haiti. In this population, the prevalence of HBsAg might have decreased from 4.8% (before 2000) to 3.1% (after 2000), whilst such decrease was not observed for the prevalence of anti-HCV antibody. The prevalence of HBsAg did not considerably vary between people living in Haiti and those who migrated to other countries; a similar pattern was observed for anti-HCV. There was no study reporting the prevalence of HDV in Haitian.

To date, multiple studies reported a country-specific estimates for HBsAg in Haiti with marked variations. The highest estimate was provided by Schweitzer et al. (7) (13.6%, 95% CI: 9.0–19.9). That systematic review and meta-analysis only included two studies with a total of 155 participants (27, 29); importantly, they used the prevalence of HBsAg in people positive for hepatitis B core antibody (anti-HBc) that had been reported in one of the two included studies (29). It is obvious that those who have been exposed to HBV (i.e., anti-HBc positive) should have higher prevalence of HBsAg than the general population, thus leading to an overestimation of the HBV burden. The pooled prevalence of HBsAg among the general adult population in our meta-analysis (3.5%, 95% CI: 2.8–4.4) fell into the range of the previous estimate reported by the Polaris Observatory Collaborators (2.9%, 95% CI: 2.7–4.1) (10). Their hybrid method combining literature review, international expert interviews, meta-analysis, and modeling, yielded a pertinent estimate because it was based on large data on blood donors and pregnant women, all included as well in this review.

The prevalence observed in our systematic review was lower than the estimates in many of Asian or African countries, where about 6% of the population carry HBsAg (47–51), but much higher than the Americas' average (0.7%, 95% CI: 0.4–1.6) (2). Assuming that 3.5% of 7.8 million adults in Haiti are chronically infected with HBV, about 273,000 people would need follow-up and care for their chronic HBV infection (52). Our findings also suggest that HBV prevalence might be higher among high-risk groups, including patients with tuberculosis or those living with HIV.

As expected, the HBsAg prevalence in pregnant women (3.8%) was similar to the prevalence in the general adult population. Although the rate of HBeAg carriage in HBsAg-positive mothers was not high, a significant proportion presented a high viral load (20.7% had a viral load ≥200,000 IU/ml). This being a strong marker for MTCT (53), it is important to consider systematic measures for prevention of MTCT of HBV in Haiti.

We found a decreasing trend of HBsAg in the general adult population by comparing the pooled estimates before and after the year 2000. This decreasing trend might be underestimated by the use of non-sensitive assay before 2000; indeed, all the pre-2000 studies but one used radioimmunoassay to detect HBsAg, which has much lower analytical sensitivity than chemiluminescence or enzyme immunoassay that were used in studies conducted after 2000. This indicates that the true prevalence of HBsAg before 2000 could have been even higher than what we observed, further confirming the decreasing trend in HBsAg prevalence over time. This spontaneous decrease in HBsAg prevalence in adults, in the absence of a large impact from a recently introduced hepatitis B immunization program, could be explained by changes in demographic factors, such as decrease in sibship size or increase in maternal age at first childbirth in Haiti (54). Delaying the age of marriage and childbearing may result in a decrease in HBsAg prevalence because older mothers, if chronically infected with HBV, have a higher chance of having lost HBeAg and remaining in low viral load with a decrease in risk of transmitting HBV to their babies (55–57). Moreover, the reduction in sibship size may contribute to the reduction in horizontal transmission that mainly occurs between siblings within a household (58, 59). This aligns with the lower prevalence seen among children after 2000.

All but one study constantly showed that the prevalence of anti-HCV was below 1%, which is close to the average for the Americas (0.7%, 95% CI: 06–0.8). There was no significant variation of the anti-HCV prevalence between studies conducted before 2000 (0.6%, 95% CI: 0.3–1.5) and after 2000 (1.0%, 95% CI: 0.6–1.6). No considerable variation in anti-HCV prevalence was observed by place of residence (0.9%, 95% CI: 0.5–1.5 in Haiti and 1.0%, 95% CI: 0.7–1.4 abroad). Assuming a prevalence of anti-HCV at 0.9% among adults and a proportion of 75% of anti-HCV positive to carry HCV RNA (60), there could be about 50,000 people in Haiti that are chronically infected with HCV and would benefit from curative treatment with directly acting antiviral (DAA). Most Haitians pay for medical fee out of pocket; therefore, it will help if DAAs are financed like antiretroviral drugs for HIV.

This review had a few constrains. First, in two studies reporting on HBsAg, we could not separate adult from children population (27, 28), one of them also including pregnant women (27). Similarly, for one study reporting anti-HCV antibody, adults and children were considered together (44). Those three studies were analyzed as adult population. Second, the study by Jean-Baptiste et al. and the Pan American Health Organization evaluating blood donors did not distinguish first time donors from repeat blood donors (34, 38); including regular blood donors in this group could cause an underestimation of the prevalence. Finally, we did not have enough studies to estimate prevalence of anti-HCV antibody for pregnant women, for PLHIV or to estimate the prevalence of HBsAg in children born after 2012.

In conclusion, this systematic review pooled prevalence for HBsAg and anti-HCV antibody among Haitian people. High prevalence of HBsAg in this population requires an urgent intervention such as increase in coverage of infant hepatitis B immunization, integration of birth dose vaccines, and prevention of mother-to-child transmission of HBV by antenatal screening and provision of peripartum antiviral prophylaxis. The prevalence of anti-HCV antibody was not as high as HBsAg, but still requires an immediate intervention as thousands of people could avoid having liver-related morbimortality in Haiti. This systematic review also identified an important knowledge gap; the prevalence of HDV needs to be evaluated in Haiti.

## Data availability statement

The original contributions presented in the study are included in the article/Supplementary material, further inquiries can be directed to the corresponding authors.

## Author contributions

JV and YS developed the study protocol and wrote the manuscript. JV performed the search and data analysis. JV and CN screened the articles and extracted the data. SW provided data. YM provided statistical support. All authors reviewed and agreed with the final version of the manuscript.
