# Supplementary material for: Prevalence of hepatitis B, C, and D virus infection in Haiti: A systematic review and meta-analysis
Source: Front Public Health. 2023 Jan 11;10:1099571. doi: 10.3389/fpubh.2022.1099571 (PMC9874305; doi:10.3389/fpubh.2022.1099571)
Supplement: Supplementary file 1 [file Data_Sheet_1.docx]

**Supplementary material**

**PubMed search strategy :**

First concept: "Haiti"[MeSH Terms] OR "Haiti*"[All Fields]

Second concept : "hepatitis b"[MeSH Terms] OR "hepatitis b virus"[MeSH Terms] OR "hepatitis b surface antigens"[MeSH Terms] OR "hepatitis b"[Text Word] OR "type b hepatitis"[Text Word] OR "hepatitis type b"[Text Word] OR "hbv"[Text Word] OR "hep b"[Text Word] OR "hbsag"[Text Word] OR "hbs-ag"[Text Word] OR "hbs antigen*"[Text Word] OR "vhb"[Text Word]

Third concept : "hepatitis c"[MeSH Terms] OR "hepacivirus"[MeSH Terms] OR "hepatitis c antibodies"[Mesh Terms] OR "hepatitis c"[Text Word] OR "type c hepatitis"[Text Word] OR "hepatitis type c"[Text Word] OR "hcv"[Text Word] OR "hep c"[Text Word] OR "vhc"[Text Word]

Fourth concept : "hepatitis d"[MeSH Terms] OR "hepatitis delta Virus"[MeSH Terms] OR "hepatitis d"[Text Word] OR "type d hepatitis"[Text Word] OR "hepatitis type d"[Text Word] OR "hepatitis delta"[Text Word] OR "delta hepatitis"[Text Word] OR "delta virus"[Text Word] OR "hdv"[Text Word] OR "hep d"[Text Word] OR "vhd"[Text Word]

First AND (Second OR Third OR Fourth) were used.

**Supplementary Figure 1.** **Prevalence of HBsAg among people living with HIV**

**
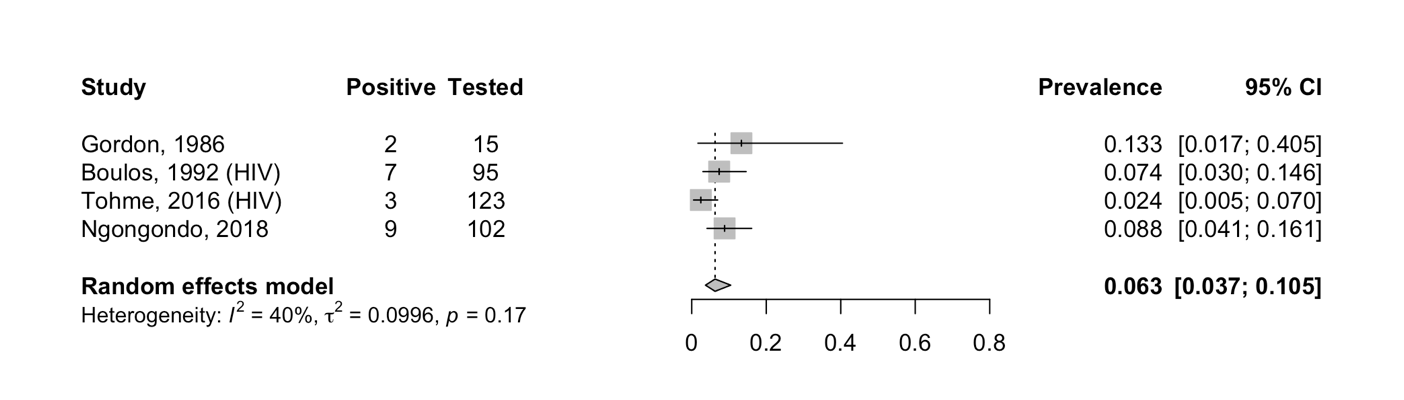
**

(HIV), human immunodeficiency virus positive group.

**Supplementary Figure 2. Prevalence of HBeAg among HBsAg positive mothers**


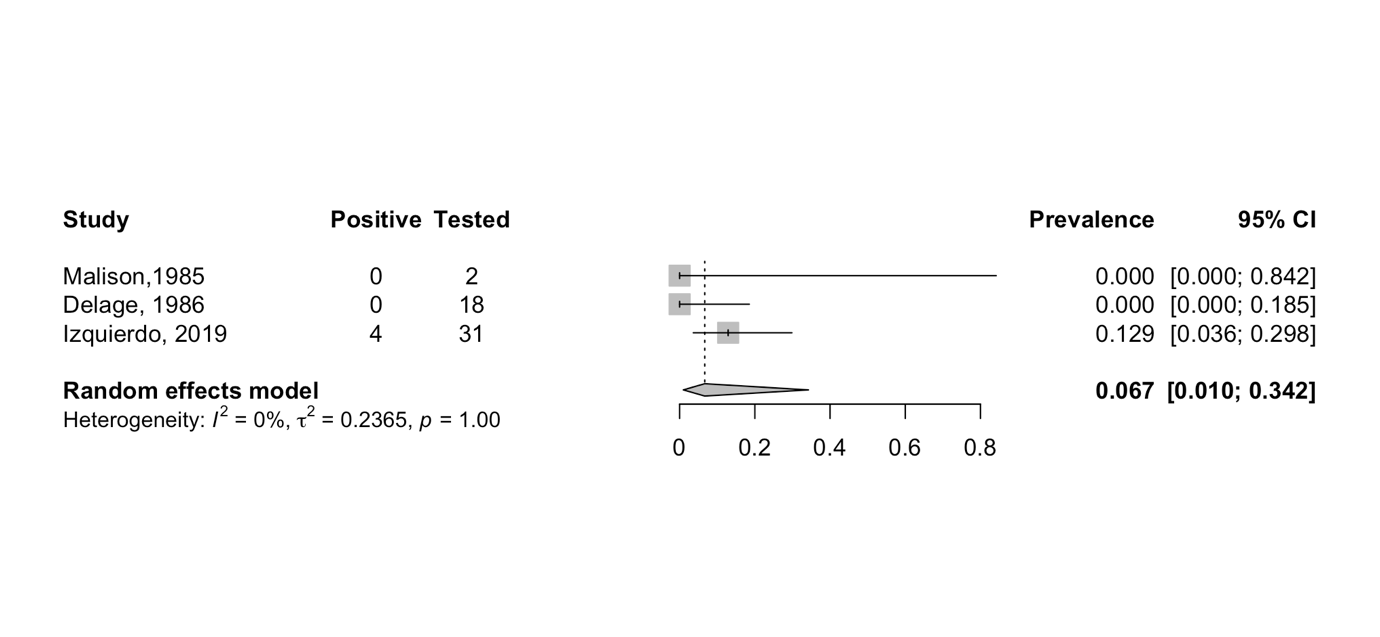


Supplementary Table 1. Risk of bias of studies reporting HBsAg prevalence

|  |  |  | External Validity | | | | Internal Validity | | |  |  |
| --- | --- | --- | --- | --- | --- | --- | --- | --- | --- | --- | --- |
| ID | **Author** | **Publication Year** | **1.Was the study’s target population a close representation of the national population in relation to relevant variables?**   - **Yes: Low** - **No: High** | **2. ﻿Was the sampling frame a true or close representation of the target population?**   - **Community-based: Low** - **Hospital-based: High** | **3. Was some form of random selection used to select the sample, OR was a census undertaken?**   - **Yes: Low** - **No: High** | **4. Was the likelihood of nonresponse bias minimal?i.e. number elligible for testing close to number tested.**   - **Yes, ≥80% of eligible were screened: Low** - **No, <80% of eligible were screened: High** | **5.a. Was the same type of test used for all subjects?**   - **Yes: Low** - **No: High** | **5.b. Was a highly sensitive assay used?**   - **Yes, enzyme immunoassay (EIA) or chemiluminescent immunoassay (CIA): Low** - **No, radioimmunoassay (RIA) or haemagglutination assay (HA) or immunochromatography (IC): High** | **6. Were the numerator(s) and denominator(s) for the parameter of interest appropriate?**   - **Yes: Low** - **No: High** | **7. Summary item on the overall risk of study bias? (low, moderate, or high risk of bias)** |  |
| 1 | Jean-Baptiste | 2018 | Yes, the study was conducted on blood donors nationwide (LOW RISK) | Community (LOW RISK) | No random selection but all blood donations (LOW RISK) | Yes, 100% screened (LOW RISK) | Yes (LOW RISK) | EIA (LOW RISK) | No, the numerator was derived based on denominator and % positive (HIGH RISK) | **LOW RISK** |  |
| 2 | Gordon | 1986 | No, immigrants with HIV (HIGH RISK) | Hospital (HIGH RISK) | No, consecutive records review (LOW RISK) | Yes, 100% screened (LOW RISK) | Yes (LOW RISK) | RIA (HIGH RISK) | Yes (LOW RISK) | **HIGH RISK** |  |
| 3 | Izquierdo | 2019 | No, immigrant pregnant women (HIGH RISK) | This study recruited women with high-risk pregnancy  (HIGH RISK) | No, consecutive sampling (LOW RISK) | Unclear (UNCLEAR RISK) | Yes (LOW RISK) | CLIA (LOW RISK) | Yes (LOW RISK) | **HIGH RISK** |  |
| 4 | Andernach | 2009 | No, nationwide but only pregnant women (HIGH RISK) | Prenatal clinic is alike community, not necessarily sick attendant  (LOW RISK) | Unclear (UNCLEAR RISK) | Yes, 100% screened (LOW RISK) | Yes (LOW RISK) | EIA (LOW RISK) | No, the numerator was derived based on denominator and % positive (HIGH RISK) | **LOW RISK** |  |
| 5 | Boulos | 1992 | No, pregnant women at a clinic (HIGH RISK) | Prenatal clinic is alike community, not necessarily sick attendant  (LOW RISK) | No, case (HIV) and control selection (HIGH RISK) | No, 3% screened (HIGH RISK) | Yes (LOW RISK) | RIA (HIGH RISK) | Yes (LOW RISK) | **HIGH RISK** |  |
| 6 | Childs | 2019 | Yes, children nationwide (LOW RISK) | Yes (LOW RISK) | Yes (LOW RISK) | Yes, 98% screened (LOW RISK) | Yes (LOW RISK) | IC (HIGH RISK) | Yes (LOW RISK) | **LOW RISK** |  |
| 7 | Delage | 1986 | No, immigrant pregnant women (HIGH RISK) | Prenatal clinic is alike community, not necessarily sick attendant  (LOW RISK) | No, consecutive sampling (LOW RISK) | Unclear (UNCLEAR RISK) | 2 types of test (HIGH RISK) | RIA/EIA (HIGH RISK) | Yes (LOW RISK) | **HIGH RISK** |  |
| 8 | Exantus | 2010 | No, children from a hospital (HIGH RISK) | Hospital (HIGH RISK) | No, consecutive records review (LOW RISK) | No, 20% screened (HIGH RISK) | Unclear (UNCLEAR RISK) | N/R (UNCLEAR RISK) | Yes (LOW RISK) | **HIGH RISK** |  |
| 9 | Fuster | 2020 | No, immigrants (HIGH RISK) | Community (LOW RISK) | No, convenience sampling (HIGH RISK) | Yes, 94% screened (LOW RISK) | Yes (LOW RISK) | CMIA (LOW RISK) | No, the numerator was derived (HIGH RISK) | **MODERATE RISK** |  |
| 10 | Rosenblum | 1990 | No, immigrants (HIGH RISK) | Community (LOW RISK) | Yes (LOW RISK) | Yes, 94% screened (LOW RISK) | Yes (LOW RISK) | RIA (HIGH RISK) | Yes (LOW RISK) | **LOW TO MODERATE RISK** | |
| 11 | Brogden | 2021 | No, immigrants (HIGH RISK) | Hospital (mainly) (HIGH RISK) | No, consecutive sampling: automaticaly captured by country of birth in ER (LOW RISK) | Yes, 82% screened (LOW RISK) | Yes (LOW RISK) | EIA (LOW RISK) | Yes (LOW RISK) | **MODERATE RISK** |  |
| 12 | Franke | 2021 | Not clear (UNCLEAR RISK) | Hospital (HIGH RISK) | No, consecutive sampling (LOW RISK) | No, <50% screened (HIGH RISK) | Yes (LOW RISK) | IC (HIGH RISK) | Yes (LOW RISK) | **HIGH RISK** |  |
| 13 | Ngongondo | 2018 | Not clear (UNCLEAR RISK) | Hospital (HIGH RISK) | Yes (LOW RISK) | Yes, 93.6% screened (LOW RISK) | N/R (UNCLEAR RISK) | N/R (UNCLEAR RISK) | Yes (LOW RISK) | **HIGH RISK** |  |
| 14 | Jones | 2021 | No, immigrants (HIGH RISK) | Community (LOW RISK) | No, convenience sampling (HIGH RISK) | Yes, 88% screened (LOW RISK) | Yes (LOW RISK) | EIA (LOW RISK) | Yes (LOW RISK) | **MODERATE RISK** |  |
| 15 | Tohme | 2016 | No, nationwide but only pregnant women (HIGH RISK) | Community (LOW RISK) | Yes (LOW RISK) | Yes, 96% screened (LOW RISK) | Yes (LOW RISK) | CLIA (LOW RISK) | Yes (LOW RISK) | **LOW RISK** |  |
| 16 | PAHO | 2020 | Yes, the study was conducted on blood donors nationwide (LOW RISK) | Community (LOW RISK) | No random selection but all blood donations (LOW RISK) | Yes, 100% screened (LOW RISK) | N/R (UNCLEAR RISK) | N/R (UNCLEAR RISK) | No, the numerator was derived based on denominator and % positive (HIGH RISK) | **LOW RISK** |  |
| 17 | Malison | 1985 | No, immigrants (HIGH RISK) | Community (LOW RISK) | Yes (LOW RISK) | Not clear (UNCLEAR RISK) | Yes (LOW RISK) | RIA (HIGH RISK) | Yes (LOW RISK) | **MODERATE RISK** |  |
| 18 | Rein | 2010 | No, refugees (HIGH RISK) | Immigration centers (HIGH RISK) | No, consecutive sampling (LOW RISK) | Data from 31/47 states (HIGH RISK) | Not clear (UNCLEAR RISK) | Not clear (UNCLEAR RISK) | Yes (LOW RISK) | **HIGH RISK** |  |
| 19 | Jonas | 1989 | No, immigrants (HIGH RISK) | Maternity ward (LOW RISK) | No, consecutive sampling (LOW RISK) | Yes, 100% screened (LOW RISK) | Yes (LOW RISK) | RIA (HIGH RISK) | Yes (LOW RISK) | **MODERATE RISK** |  |
| 20 | Ollé-Goig | 1985 | No, patients at one hospital (HIGH RISK) | Hospital (HIGH RISK) | No, case (ascites) and control selection (HIGH RISK) | Yes, 100% screened (LOW RISK) | Yes (LOW RISK) | RIA (HIGH RISK) | Yes (LOW RISK) | **MODERATE RISK** |  |
| 21 | Lange | 1988 | No, refugees (HIGH RISK) | Immigration centers (HIGH RISK) | Not clear (UNCLEAR RISK) | Not clear (UNCLEAR RISK) | Yes (LOW RISK) | RIA (HIGH RISK) | Yes (LOW RISK) | **HIGH RISK** |  |
| 22 | Schill | 1989 | No, patients and pregnant women at a rural hospital (HIGH RISK) | Hospital (HIGH RISK) | Yes (LOW RISK) | Yes, 100% screened (LOW RISK) | Yes (LOW RISK) | EIA (LOW RISK) | Yes (LOW RISK) | **MODERATE RISK** |  |

Supplementary Table 2. Risk of bias of studies reporting anti-HCV antibody prevalence

|  |  |  | External Validity | | | | | | Internal Validity | | | |  | |
| --- | --- | --- | --- | --- | --- | --- | --- | --- | --- | --- | --- | --- | --- | --- |
| ID | **Author** | **Publication Year** | **1. Was the study’s target population a close representation of the national population in relation to relevant variables?**   - **Yes: Low** - **No: High** | **2. ﻿Was the sampling frame a true or close representation of the target population ?**   - **Community-based: Low** - **Hospital-based: High** | **3. Was some form of random selection used to select the sample, OR was a census undertaken?**   - **Yes: Low** - **No: High** | | **4. Was the likelihood of nonresponse bias minimal?i.e. number elligible for testing close to number tested.**   - **Yes, ≥80% of eligible were screened: Low** - **No, <80% of eligible were screened: High** | | **5.a. Was the same type of test used for all subjects?**   - **Yes: Low** - **No: High** | **5.b. Was a highly sensitive assay used?**   - **Yes, enzyme immunoassay (EIA) or chemiluminescent immunoassay (CIA): Low** - **No, radioimmunoassay (RIA) or haemagglutination assay (HA) or immunochromatography (IC): High** | **6. Were the numerator(s) and denominator(s) for the parameter of interest appropriate?**   - **Yes: Low** - **No: High** | | **7. Summary item on the overall risk of study bias? (low, moderate, or high risk of bias)** | |
| 1 | Jean-Baptiste | 2018 | Yes, the study was conducted on blood donors nationwide (LOW RISK) | Community (LOW RISK) | No random selection but all blood donations (LOW RISK) | | Yes, 100% screened (LOW RISK) | | Yes (LOW RISK) | EIA (LOW RISK) | No, the numerator was derived based on denominator and % positive (HIGH RISK) | | **LOW RISK** | |
| 9 | Fuster | 2020 | No, immigrants (HIGH RISK) | Community (LOW RISK) | No, convenience sampling (HIGH RISK) | | Yes, 94% screened (LOW RISK) | | Yes (LOW RISK) | CMIA (LOW RISK) | No, the numerator was derived (HIGH RISK) | | **MODERATE RISK** | |
| 11 | Brogden | 2021 | No, immigrants (HIGH RISK) | Hospital (mainly) (HIGH RISK) | No, consecutive sampling: automaticaly captured by country of birth in ER (LOW RISK) | | Yes, 82% screened (LOW RISK) | | Yes (LOW RISK) | EIA (LOW RISK) | Yes (LOW RISK) | | **MODERATE RISK** | |
| 12 | Franke | 2021 | Not clear (UNCLEAR RISK) | Hospital (HIGH RISK) | No, consecutive sampling (LOW RISK) | | No, <50% screened (HIGH RISK) | | Yes (LOW RISK) | IC (HIGH RISK) | Yes (LOW RISK) | | **HIGH RISK** | |
| 14 | Jones | 2021 | No, immigrants (HIGH RISK) | Community (LOW RISK) | No, convenience sampling (HIGH RISK) | | Yes, 93% screened (LOW RISK) | | Yes (LOW RISK) | EIA (LOW RISK) | Yes (LOW RISK) | | **MODERATE RISK** | |
| 16 | PAHO | 2020 | Yes, the study was conducted on blood donors nationwide (LOW RISK) | Community (LOW RISK) | No random selection but all blood donations (LOW RISK) | Yes, 100% screened (LOW RISK) | | N/R (UNCLEAR RISK) | | N/R (UNCLEAR RISK) | | No, the numerator was derived based on denominator and % positive (HIGH RISK) | **LOW RISK** |  |
| 23 | Hepburn | 2004 | No, outpatients with laboratory tests at one hospital (HIGH RISK) | Hospital (HIGH RISK) | No, consecutive sampling (LOW RISK) | | Yes, 100% screened (LOW RISK) | | Yes (LOW RISK) | EIA (LOW RISK) | Yes (LOW RISK) | | **MODERATE RISK** | |
| 24 | Talarmin | 1997 | No, immigrants (HIGH RISK) | Community (LOW RISK) | Yes (LOW RISK) | | Yes (LOW RISK) | | Yes (LOW RISK) | EIA (LOW RISK) | Yes (LOW RISK) | | **LOW TO MODERATE RISK** | |
| 25 | Allain | 1992 | No, outpatients, surgery patients and pregnant women at a rural hospital (HIGH RISK) | Hospital (HIGH RISK) | No, convenience sampling (HIGH RISK) | | No, 38% screened (HIGH RISK) | | Yes (LOW RISK) | EIA (LOW RISK) | Yes (LOW RISK) | | **MODERATE RISK** | |
